# ‘Care for Stroke’, a web-based, smartphone-enabled educational intervention for management of physical disabilities following stroke: feasibility in the Indian context

**DOI:** 10.1136/bmjinnov-2015-000056

**Published:** 2015-07

**Authors:** K Sureshkumar, G V S Murthy, Suresh Munuswamy, Shifalika Goenka, Hannah Kuper

**Affiliations:** 1 International Centre for Evidence in Disability, London School of Hygiene and Tropical Medicine, London, UK; 2 Institute of Public Health-Hyderabad, Hyderabad, Telangana, India; 3 Institute of Public Health-Delhi, Gurgaon, Haryana, India

**Keywords:** Geriatrics, Global Health, mHealth, Neurology, Assistive Technology

## Abstract

**Introduction:**

Stroke rehabilitation is a process targeted towards restoration or maintenance of the physical, mental, intellectual and social abilities of an individual affected by stroke. Unlike high-income countries, the resources for stroke rehabilitation are very limited in many low-income and middle-income countries (LMICs). Provision of cost-effective, post-stroke multidisciplinary rehabilitation services for the stroke survivors therefore becomes crucial to address the unmet needs and growing magnitude of disability experienced by the stroke survivors in LMICs. In order to meet the growing need for post-stroke rehabilitation services in India, we developed a web-based Smartphone-enabled educational intervention for management of physical disabilities following a stroke.

**Methods:**

On the basis of the findings from the rehabilitation needs assessment study, guidance from the expert group and available evidence from systematic reviews, the framework of the intervention content was designed. Web-based application designing and development by Professional application developers were subsequently undertaken.

**Results:**

The application is called ‘Care for Stroke’. It is a web-based educational intervention for management of physical disabilities following a stroke. This intervention is developed for use by the Stroke survivors who have any kind of rehabilitation needs to independently participate in his/her family and social roles.

**Discussion:**

‘Care for stroke’ is an innovative intervention which could be tested not just for its feasibility and acceptability but also for its clinical and cost-effectiveness through rigorously designed, randomised clinical trials. It is very important to test this intervention in LMICs where the rehabilitation and information needs of the stroke survivors seem to be substantial and largely unmet.

## Background

Stroke rehabilitation is a process targeted towards restoration or maintenance of the physical, mental, intellectual and social abilities of an individual affected by stroke.^1^ Stroke rehabilitation enables the stroke survivor to perform his/her daily activities at an optimal functional level and helps the stroke survivor to participate in his/her social roles as independently as possible.^2^ The stroke survivor relearns the skills that are lost or impaired due to brain damage following stroke through rehabilitation.^3^

An insult to the human brain due to stroke might have various effects on the stroke survivor, and hence healthcare professionals from various disciplines have to provide the stroke survivor with a patient-centred, comprehensive, multidisciplinary rehabilitation.^4^ Unlike high-income countries (HICs), the resources for rehabilitation, especially the rehabilitation workforce and infrastructure, are very limited in many low and middle-income countries (LMICs).^5^ If we take India as an example, rehabilitation services are often unidisciplinary, driven predominantly by physiotherapists, with lack of support from occupational therapists, speech therapists and so on. Many government-run district rehabilitation centres are non-functional and the private hospitals are staffed with only a physiotherapist in their rehabilitation centres.^6^ Given the scarce resources, the rehabilitation needs of the stroke survivors, especially in the LMICs, remain largely unmet.^7^ Provision of cost-effective, post-stroke multidisciplinary rehabilitation services for the stroke survivors therefore becomes crucial to address the unmet needs and growing magnitude of disability experienced by the stroke survivors in LMICs.

The past few years have seen a tremendous increase in the use of Smartphones by health professionals and also by the general public.^8^ Evidence from a recent systematic review suggests that Smartphones could be an extremely useful tool to educate patients to manage their health problems.^9^ Another systematic review on the use of Smartphone applications for stroke rehabilitation also demonstrates the advantages of Smartphone applications for provision of stroke-related information.^10^ These Smartphone applications are regarded as important by health professionals providing stroke rehabilitation themselves.^10^

In order to meet the growing need for post-stroke rehabilitation services in India, we developed a web-based Smartphone-enabled educational intervention for management of physical disabilities following stroke. This paper provides a detailed description of the intervention and the processes involved in its development. The paper also discusses the importance of such rehabilitation interventions for meeting the unmet needs of the stroke survivors.

## Development of the content for the intervention

### Systematic review of the available interventions

Evidence from systematic reviews in relation to stroke rehabilitation and information provision for stroke survivors and caregivers was extensively used to develop the intervention. We also conducted a comprehensive and a global systematic review on educational interventions for reducing disabilities in acquired brain injury to investigate the evidence that was available to develop this intervention.

### Rehabilitation needs assessment study

The content of the intervention was developed primarily based on the needs expressed by the stroke survivors and caregivers who participated in a rehabilitation needs assessment study carried out exclusively to develop this intervention. The rehabilitation needs assessment study was carried out to guide the development of a need-based rehabilitation intervention and had two components in it. One was a structured survey with 50 stroke survivors and their caregivers to identify the various kinds of rehabilitation needs that they experience. The other was a detailed in-depth interview with a subsample of the stroke survivors and caregivers selected for the survey. The purpose of the in-depth interviews was to gain a detailed understanding of the experiences of the stroke survivors in relation to accessing stroke rehabilitation services and their rehabilitation needs following a stroke. In-depth interviews with health professionals involved in the provision of stroke rehabilitation services were also carried out to understand the perspective of the health professionals about provision of stroke rehabilitation services, their knowledge about the existing Smartphone-based health interventions and their attitudes and opinions about the use of a Smartphone-enabled, care-supported education programme for domiciliary stroke rehabilitation.

### Expert group for content development

In addition to the needs assessment, expert guidance was obtained from a team of eight highly qualified and experienced health professionals from various neurorehabilitation disciplines (physical medicine and rehabilitation, neuropsychiatry, clinical psychology, occupational therapy, physiotherapy, social sciences, information technology, public health and m-health) with both national and global expertise in the field of neuropsychiatric rehabilitation. The expert team also included three stroke survivors and their primary caregivers. All the team members were from Tamil Nadu and they were Tamil-speaking. The key characteristics of the expert group, such as their experience, expertise, global exposure and language, facilitated the development of a culturally specific, patient-centred intervention for management of physical disabilities following a stroke.

### Framework of the intervention content

On the basis of the findings from the rehabilitation needs assessment study, guidance from the expert group, and available evidence from systematic reviews, the framework of the intervention content was designed. The content framework included five important sections related to post-stroke rehabilitation. The sections were:

1. Information about stroke (know more about stroke)
2. Exercises (home-based exercises)
3. Functional skills training (preparing oneself for daily living)
4. Activities of daily living (engaging in activities of daily living)
5. Assistive devices (devices to assist daily living).

## Content of the intervention sections

### Know more about stroke

As the section title suggests, this section enables the stroke survivors and caregivers to know more about stroke, the impact of stroke on an individual experiencing it and advice from experts on the way forward (life after a stroke). The important subsections/topics and videos that this section includes are provided in table 1.

**Table 1:** Content of ‘know more about stroke’ section

Content of the intervention		
Main sections	Subsections	Videos
Information about stroke	What is a stroke? What is a transient ischaemic attack (TIA)? How does a stroke happen? Warning signs of a stroke What are the common symptoms of a stroke? How does a stroke affect your body? Risk factors for a stroke Common effects of a stroke Recovering from a stroke	What is a stroke? How does a stroke happen? What is a transient ischaemic attack Symptoms of a stroke Effects of a stroke Modifiable and non-modifiable risk factors for a stroke Effects of a stroke on Balance Bowel and Bladder Thinking Pain Physical problems Sleep and fatigue Sensation Sleep and fatigue Speech and language Swallowing Recovery from a stroke by Public health experts a neuropsychiatrist a neurologist a physiotherapist an occupational therapist a clinical psychologist a disability rights expert

The primary objective of having this section is to create awareness and enable the stroke survivors and their caregivers to gain more knowledge about stroke, because this would assist them in preventing recurrent stroke, modifying their lifestyle, making treatment decisions and planning for life after a stroke.

### Home-based exercises

This section includes home-based, task-oriented exercises that the stroke survivors can practise in their home in order to maintain or improve their body fitness for functional activities. These exercises are based on eclectic treatment approaches to stroke rehabilitation (motor relearning, functional, neurodevelopmental frame of references for therapy) that enable the stroke survivors to use their affected parts of the body and engage in functional activities.

These home-based exercises include the use of equipment like a chair or bed and table that are commonly available in most homes in India. They do not require the purchase of any sophisticated exercise equipment. Principles of safety and risk/hazard prevention have been thoroughly considered while developing this section. Some of the important subsections/topics and videos that this section comprises are listed in table 2.

**Table 2:** Content of ‘Exercises’ section

Content of the intervention		
Main sections	Subsections	Videos
Exercises	Upper limb exercises Lower limb exercises Balance exercises Active exercises Exercises to improve upper limb function	Passive upper limb exercises Passive lower limb exercises Active-assisted exercises for the lower limb Active exercises for the upper limb Exercises for the trunk Exercises for balance Improving awareness and function of the affected hand

The objective of developing this section content is to enable the stroke survivors to understand the relevance of the conscious use of the affected parts of the body following a stroke and also the importance of exercises for engaging in functional activities rather than just exercising and improving the flexibility, strength and movement of the affected body part.

### Preparing oneself for everyday living

Functional skills are a prerequisite to participate in everyday living. One should know how to get up from a lying down position. In order to sit properly and feed or groom, one should know how to transfer from a bed to a chair or a commode for bathing or toileting. These are very important to the stroke survivor who cannot or finds it difficult to move the affected part of his/her body. This section highlights functionally oriented tasks that the stroke survivors can learn in order to participate in their day-to-day activities.

Exercise training provided by a physiotherapist to the stroke survivors is directly related to the development of functional skills of the individual affected by stroke. Hence, this section stresses the importance of functional skills to participate in everyday living and preparing oneself for everyday living by acquiring functional skills. Some of the important subsections/topics and videos of this section are depicted in table 3.

**Table 3:** Content of ‘Exercises’ section

Content of the intervention		
Main sections	Subsections	Videos
Functional skills training	Positioning the stroke survivor in bed and in a chair Bed mobility Transfers Standing up from a sitting Mobility/ambulation training	Positioning on the Chair – the Bed –affected side the Bed –unaffected side the Bed—Lying on the back Bed Mobility Rolling on the bed Scooting on the bed Coming up to a sitting Sit to Stand (moderate support) Transfers Independent transfers (bed to chair/wheelchair) Transfers with maximum support Walking

### Engaging in activities of daily living

This section comprises adaptive methods and techniques to engage in activities of daily living like grooming, bathing, dressing and eating. The stroke survivors can watch, learn and practise these adaptive techniques to independently perform their activities of everyday living. The content of this section is depicted in table 4.

**Table 4:** Content of ‘Activities of daily living’ section

Content of the intervention		
Main sections	Subsections	Videos
Activities of daily living	Brushing Feeding Bathing Grooming Dressing	Brushing Feeding Bathing Grooming Washing face Wearing a T-shirt Wearing a Shirt Wearing a dhoti/lungi Wearing a pant Wearing a saree Wearing a blouse Undressing

This section is very important from the viewpoint of both the stroke survivors and their caregivers. This is because learning to purposefully engage in one's own everyday living seems to be an important need and crucial task for the stroke survivors to independently participate in his/her personal, family and societal roles. Although the stroke survivors learn to do exercises and acquire knowledge to manage their problems post-stroke, the overall objective behind the acquisition of these skills and knowledge is to live a functionally independent life and perform their various roles at home and society actively (table 5).

**Table 5:** Content of ‘Devices to assist daily living’ section

Content of the intervention		
Main sections	Subsections	Videos
Assistive devices	Personal care aids Mobility aids Orthoses and supports	Personal care aids Mobility aids Orthoses and supports

### Devices to assist daily living

This is a unique section that enables the stroke survivors and their caregivers to understand the importance of using assistive devices that are readily available in India and that can assist the stroke survivor to engage in their day-to-day activities independently and also with confidence. This section also includes devices that are tailor-made to the needs of the stroke survivors living in the southern part of India like an adapted saree, Velcro-based blouse, adapted dhoti and lungi, etc. This section also has devices that are not available in India but can be designed and fabricated by the stroke survivors themselves and their caregivers, for example, the universal cuff that can assist the stroke survivor to use their affected hand for feeding, brushing, writing and grooming. The key topics covered under this section is provided in table 5.

The primary objective of this section is to inform the stroke survivors of the importance of assistive devices that can be used to perform everyday activities independently and safely. Assistive devices can boost the confidence of the stroke survivor to engage in their everyday tasks. It also reduces the assistance and support provided by the caregivers, thereby reducing the physical strain in providing care and support for the stroke survivor in their daily living tasks.

## Description of the intervention

### Naming the application

This application was intended to educate the stroke survivors and their caregivers to manage their physical disabilities following stroke. Therefore, the web-based application was named ‘**Care for Stroke**’ to emphasise the importance of enhancing the life of individuals experiencing stroke and continuum of care that is essential for a stroke survivor.

### Logo and tagline of the application

**Figure 1:**
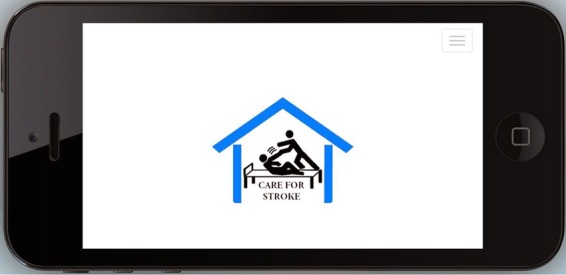
Logo of the application.

The logo of the application was created by the principal investigator himself under the supervision of experts from the field of disability, rehabilitation and design (figure 1). The logo depicts a stroke survivor accepting support from another person in a home environment and trying to mobilise himself/herself. The design of the logo stresses the importance of the stroke survivor accepting support from another person and actively engaging in functional activities while staying at home.

The tagline of the application is **‘Think Smart—Take Control’**. This tagline emphasises the importance of proactive, innovative and smart planning for therapy and rehabilitation services that the stroke survivor and their caregivers should execute, outside the hospital environment. It also encourages the stroke survivors to take control of their problems following stroke and work towards an independent life after a stroke.

### Design of the web-based application

**Figure 2:**
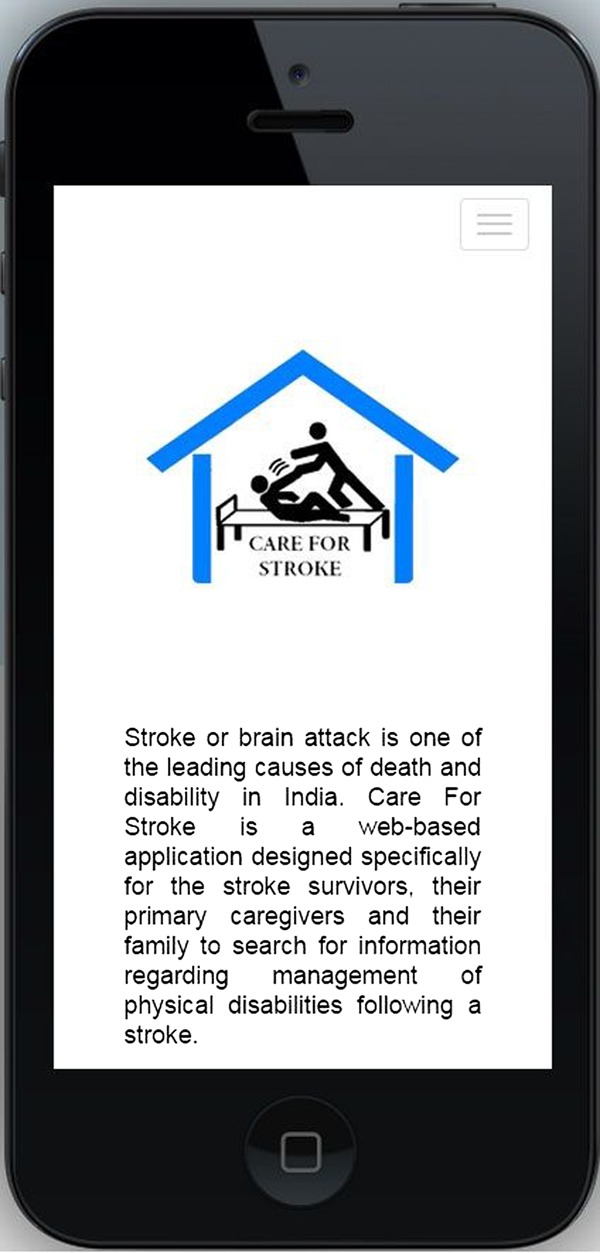
Introductory page of the ‘care for stroke’ application.

This intervention is designed as a web-based application that uses a website as an interface (the front end). The introductory web page of the application is shown in figure 2 Users can access the application not just from Smartphones but also from a computer, PDA, Tablet and even digital television that is connected to the internet using any standard web browser. Some of the key design features of this application are: User interface, content format, language.

#### User interface

An interface enables a user to interact with a system (Smartphone in this instance) to perform a task. For example: Navigating to different web pages in this website enables a user to find the video content that he/she prefers to watch. The users can watch the videos by navigating through user-friendly interfaces such as the touch and slide option which requires the users to either touch or slide the icons (ie, pictures and symbols) and pages in the application to watch the videos they want.

#### Content format

This application is exclusively designed to support digitised audio–visual content. More than 75% of the content of this application is in the form of videos. The users can interact with the images related to the main sections and watch the videos about stroke and the management of physical disability post-stroke through this application. There is very minimal requirement for the users to read written information in this application.

#### Language

This application is built with multilingual functionality and it currently supports English and Tamil, the native language of the State of Tamil Nadu in India where it was piloted.

### Technical description of the application

The application is built using a LAMP (Linux, Appache, MySQL, PHP) environment. The user interface of the application was designed using HTML5, CSS3, Bootstrap, Java Script, JQuery, Ajax, Google font API and Touch Swipe. It is to ensure that the user interface acts as a responsive and interactive design. Designing the application with these technologies supports the application to be installed and run on multiple devices like desktop, laptop, IPhone, IPad, Android devices andWindows devices.

The back end of the application was built in PHP5 (PHP—Hypertext Processor) language. This is to facilitate the user to interact with the database (MySQL) and view the requested information without any difficulty. Given the issues with video streaming in a country like India (ie, very slow internet connectivity and streaming), this application uses Cloud Flare CDN (Content Delivery Network) that enhances the quality and speed of the video streaming while the user is accessing the videos from the application.

This application also has an administrator module, where the administrator can monitor all the activities of the users who have logged into the application. It can also generate different types of reports of the user interaction with the application. Some of the key information that could be monitored are:

- The title of the sections and videos viewed,
- Duration of the logged in session
- Date and time of viewing
- Number of sections and videos watched during a logged in session.
- Device used for logging in
- Time spent on application,
- Geo-location information.

## Structure and functionality of the application

### Registered website

This web-based application can be accessed from the registered website name http://www.careforstroke.com

### Home page

The application has a home page that briefly describes stroke and stroke-related disability in the vernacular (Tamil). First-time users cannot access the intervention without registering themselves. This is to ensure proactive engagement of the users, observe their utilisation pattern and to generate utilisation reports for future evaluations. The home page provides details of registration with an icon to register the first-time users. Users who have already registered to access the intervention can use the same icon to access the sign-in page (figure 2). There is a drop-down icon in the home page to change the language of the application if required. Currently, the application pages have the descriptions in English and Tamil.

### Sign-in page and registration

This page contains an icon for first-time users to register and the sign in boxes with user name and password sections to be filled by the user to sign into the application.

### Registration page

**Figure 3:**
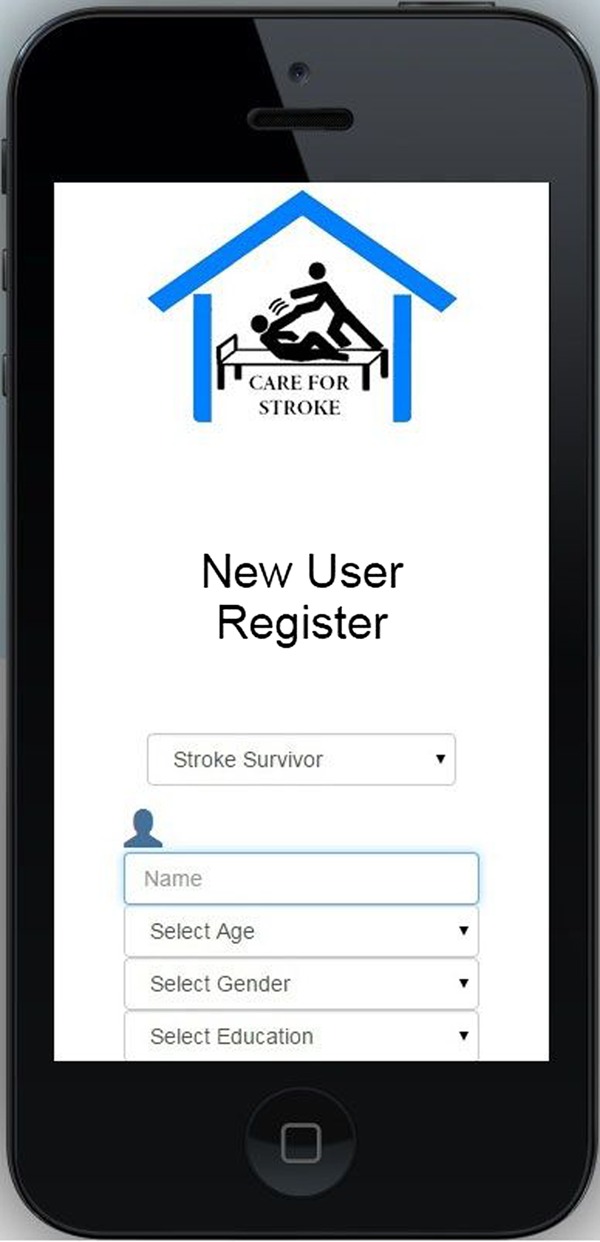
Registration page.

This page contains a drop-down box, where the user can identify and register themselves as a stroke survivor or caregiver of the stroke survivor. This helps the investigator or administrator to monitor the engagement and usage of the application by the stroke survivors and caregivers separately. On the basis of the options chosen, the user will be redirected to the specific registration page with drop-down options and text boxes to fill in the user details requested and register onto the application. After completing the registration, users will be redirected back to the sign-in page. Registration requires the users to have a username and password to ensure identity and privacy (figure 3).

### Intervention page

**Figure 4:**
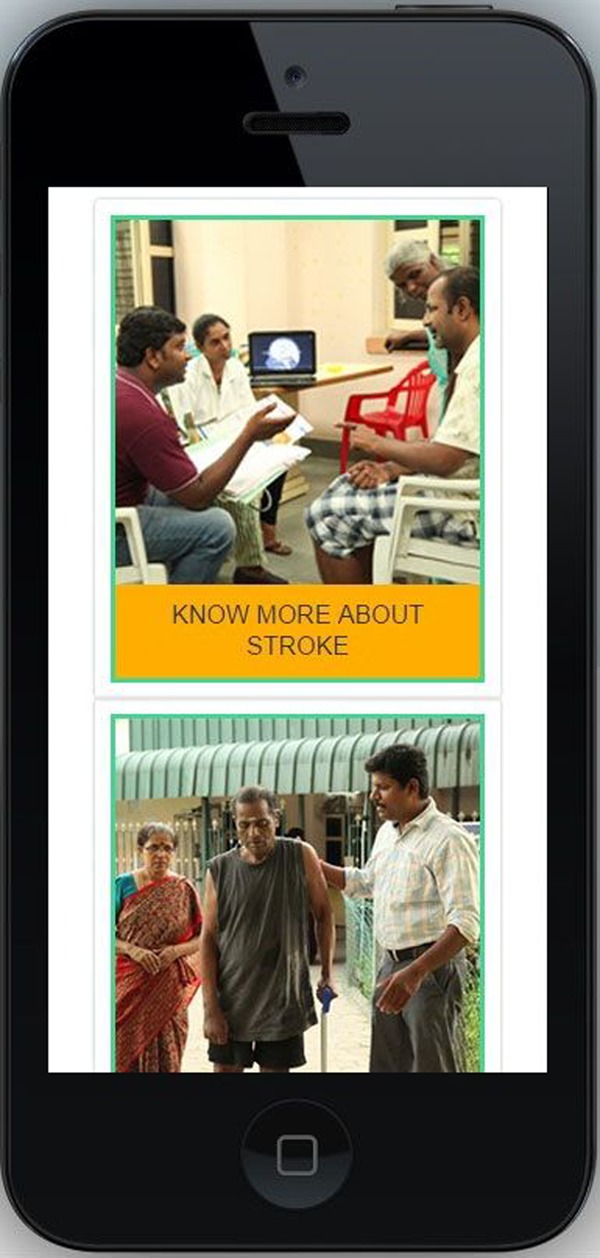
Intervention page.

After the user signs into the application successfully, the application is redirected to the main intervention page. This page contains brief written information about the intervention and five important sections that contain the content of the ‘care for stroke’ intervention (figure 4).

**Figure 5:**
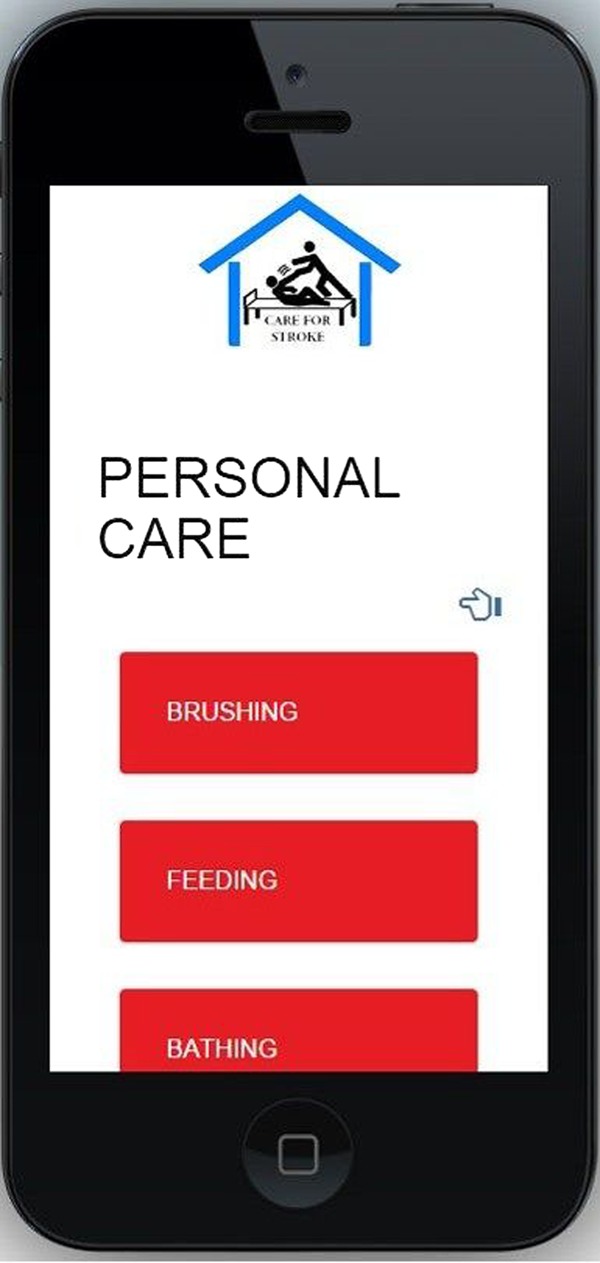
Section page.

*Sections*: There are five main sections displayed as photographic icons on the intervention page which can be touched and explored further (figure 4). These five sections contain digitised information (videos) about stroke and the various aspects that a stroke survivor can view and understand about the management of the physical disabilities following a stroke (figure 5).

**Figure 6:**
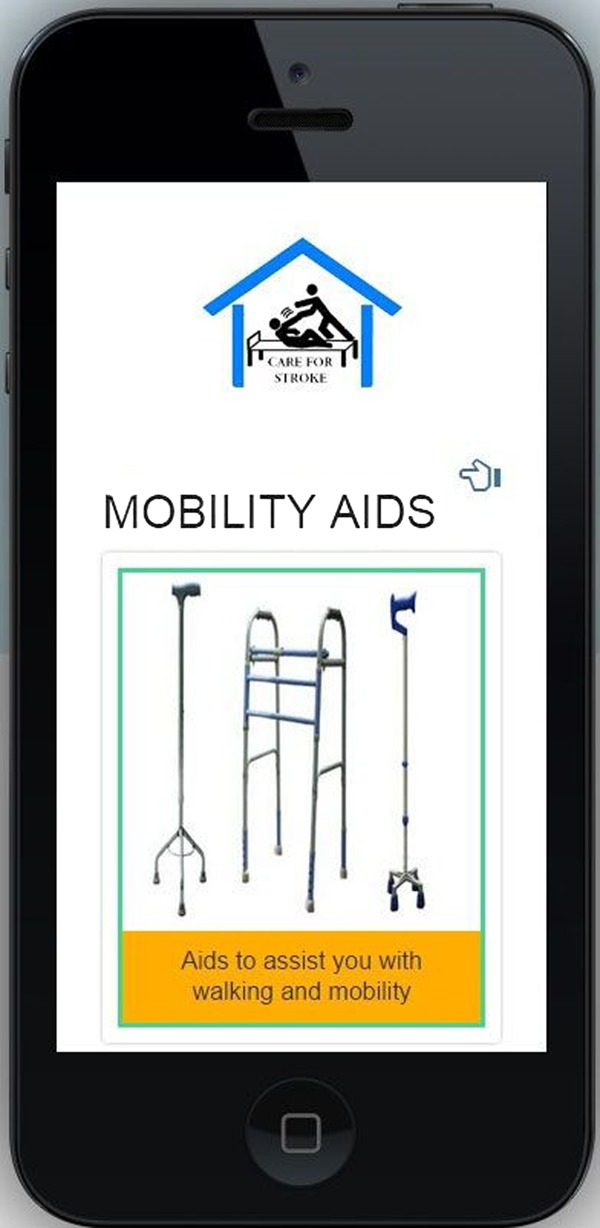
Subsection page.

*Subsections*: When the user touches an icon on the section page, it is redirected to the corresponding subsection page that comprises topics (subsections) that the respective section contains. For example, the main intervention page will contain a photograph of the stroke survivor performing his Activities of Daily Living—ADL (intervention page); if the user touches this icon, it will take him or her to the ADL section (figure 5). If the user touches this ADL section icon, the web page will be redirected to the ADL subsection page that contains topics with video icons (images) related to ADL, in this instance, stroke survivors performing brushing, feeding, dressing, etc. Please find the section web page in figure 6.

**Figure 7:**
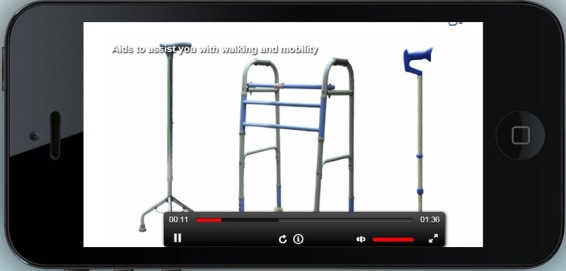
Video section of the application.

*Content digitised videos*: When the user touches a topic in the subsection, the web page will be redirected to a page that contains detailed information about that topic in the form of 3–5 min video clips. For example, if the user touches the topic ‘Wearing a blouse’, the web page will be redirected to a video clip related to that topic. These videos are streamed online through internet or mobile internet networks and can be watched by touching the play button on the video clip. Please find the video section of the application in figure 7 below.

### Shuffling between the web pages

Users can shuffle between the pages by either

1. Pressing the back button on the Smartphone
2. Pressing the back icon on the web page
3. Sliding the web pages back and forth using the touch screen option on the Smartphone.

In addition to this, the user can return to the main intervention page at any time by touching the logo which is located on top of every web page of the application.

### Administrator module

This Smartphone-enabled intervention is built with an administrator module, where the usage and utilisation patterns of this application by the users can be tracked continuously and reports can be generated to inform the feasibility of this intervention and also to monitor the progress of any programmes/research projects related to this intervention when scaled up to a larger community of stroke survivors. The administrator can also add videos onto (or remove videos from) the application as and when required, thereby customising or improvising the content of the intervention according to the needs of the users. The module is protected and strictly secured through a username and password to ensure privacy and confidentiality of the user information.

## Discussion

Stroke is one of the leading causes of death and disability worldwide.^11^ Globally, nearly six million people die from stroke each year, and much of this stroke burden is borne by LMICs.^12^ Though the primary focus of many LMICs, including India, is to prevent stroke by reducing the prevalence of its risk factors, similar attention should also be given to those who survive a stroke and are disabled post-stroke.^13^ ^14^

Unlike HICs, organised multidisciplinary rehabilitation services for stroke survivors are not available in many LMICs.^15–17^ Given the context of many LMICs with a scarce rehabilitation workforce and resources for rehabilitation, it is critically important to develop innovative post-stroke rehabilitation interventions that could address the growing magnitude of post-stroke disability and meet the rising need for rehabilitation services in these countries.

The international telecommunication union estimated that six billion people were mobile phone users during 2011 globally, which is equivalent to 87% of the world's population. This report has also documented that India is one of the top markets for Smartphone sales globally.^18^ The management of chronic diseases using Smartphone technology has been described in a recent systematic review.^19^ This review identified 15 Smartphone applications for management of chronic conditions. Out of these 15 applications, there was only one application called Mayo clinic meditation that was similar to the ‘Careforstroke’ application. The Mayo clinic application helped patients practise meditation through a 15 min training video on meditation.

Some of the Smartphone applications used in stroke rehabilitation in HICs include the Dr Droid application that helps therapists to administer and track upper limb exercises for stroke rehabilitation,^20^ the Think-FAST application that features stroke prevention information and a list of stroke unit locations in Australia^21^ and PTX, a physiotherapy exercise application for individuals with any kind of neurological conditions that includes a pictorial description of the exercises for stroke survivors.^22^ The National Institute of Clinical Excellence (NICE) guidelines for long-term stroke rehabilitation also recommend the use of Smartphones for communication problems in patients with stroke.^23^

A chronic condition like stroke requires uninterrupted therapeutic care and constant monitoring during the entire continuum of recovery.^24^ In the absence of any organised stroke care services and with the limited resources for rehabilitation, a Smartphone-enabled educational intervention for management of disabilities could be a strategy to meet the substantial rehabilitation needs of stroke survivors in India. The evidence concerning the use of Smartphones in chronic disease care in India is just emerging and the use of Smartphones in health interventions to combat diseases like diabetes, hypertension and cardiovascular diseases is progressively being investigated.^25^ Adoption of this strategy could possibly reduce the barriers to access and availability of stroke rehabilitation services. It could also aid in efficient and sustained monitoring of patient progress throughout the continuum of care.

‘Care for stroke’ is a Smartphone-enabled educational intervention for management of physical disabilities following a stroke. The content of the intervention was developed systematically and primarily based on the needs of the stroke survivors and informed by existing global evidence. It includes inputs from highly qualified and experienced multidisciplinary stroke rehabilitation professionals in a digitised audio–visual format that is more entertaining to watch and learn compared to the other methods of patient education such as an educational workbook and group teaching or lectures.

This intervention is culture-specific and language–specific, and therefore the users can easily understand and adapt the techniques to manage their post-stroke-related disabilities. Since the intervention is loaded onto a Smartphone, the user can access the intervention as and when they need. Unlike television and DVD players, Smartphones are portable and handheld and hence it might aid the user to access the intervention conveniently (without having to plug wires, operate a remote to watch videos or depend on electricity).

This Smartphone-based, technology-driven intervention can be less demanding in terms of the physical abilities required by the users to learn, when compared with other kinds of educational interventions like attending group sessions, using a stroke workbook or watching a DVD educational material about stroke. The application for accessing the intervention is web-based, and hence the users can also access the content through their laptops, desktops and tablets if required.

From the point of view of programme managers and evaluators, this kind of web-based educational intervention can continuously monitor the usage and utilisation pattern of the intervention by each user, and it can be helpful to generate reports to monitor the efficiency and effectiveness of this intervention while scaling up, without having to contact the users. Since the intervention is Smartphone-enabled and web-based, the user can contact the service provider directly by dialling the contact numbers on the Smartphone or by making a skype call using the mobile internet services.

This Smartphone-enabled intervention might also motivate the caregivers and family members to comprehend the importance of stroke rehabilitation and support the stroke survivors in utilising the key aspects of the intervention in their everyday life. From a financial perspective, the cost of using this Smartphone-enabled intervention might be less costly compared to the other ways of accessing information about stroke and the ways to manage post-stroke physical disability from rehabilitation experts or hospitals.

The ‘Care for stroke’ application is currently under pilot testing for its feasibility and acceptability with a small group of stroke survivors and their caregivers in Chennai, India. If this application is found feasible and acceptable, the investigators intend to look at the clinical and cost-effectiveness of this intervention. To date and to the best of our knowledge, there has not been a web-based, Smartphone-enabled educational application and intervention for stroke survivors with a primary focus on the rehabilitation aspect of the stroke. In a global context and from a public health perspective, ‘Care for stroke’ is one such kind of innovative intervention which could be tested not just for its feasibility and acceptability but also for its clinical and cost-effectiveness through rigorously designed, randomised clinical trials. It is very important to test this intervention in LMICs where the rehabilitation and information needs of the stroke survivors seem to be substantial and largely unmet.
